# Understanding factors associated with rural‐urban disparities of stunting among under‐five children in Rwanda: A decomposition analysis approach

**DOI:** 10.1111/mcn.13511

**Published:** 2023-03-30

**Authors:** Chester Kalinda, Million Phiri, Simona J. Simona, Andrew Banda, Rex Wong, Maria Albin Qambayot, Sage Marie Consolatrice Ishimwe, Alemayehu Amberbir, Bekele Abebe, Alemayehu Gebremariam, Julius Odhiambo Nyerere

**Affiliations:** ^1^ Bill and Joyce Cummings Institute of Global Health University of Global Health Equity Kigali Rwanda; ^2^ School of Humanities and Social Sciences University of Zambia Lusaka Zambia; ^3^ Centre for One Health University of Global Health Equity Kigali Rwanda; ^4^ Institute of Global Health Equity Research (IGHER) University of Global Health Equity Kigali Rwanda; ^5^ School of Medicine University of Global Health Equity Kigali Rwanda; ^6^ Catholic Relief Services Rwanda Country Program Kigali Rwanda; ^7^ Ignite Global Health Lab, Global Research Institute William and Mary Williamsburg Virginia USA

**Keywords:** DHS, moderate and severe stunting, Oaxaca–Blinder, Rwanda

## Abstract

Childhood stunting in its moderate and severe forms is a major global problem and an important indicator of child health. Rwanda has made progress in reducing the prevalence of stunting. However, the burden of stunting and its geographical disparities have precipitated the need to investigate its spatial clusters and attributable factors. Here, we assessed the determinants of under‐5 stunting and mapped its prevalence to identify areas where interventions can be directed. Using three combined rounds of the nationally representative Rwanda Demographic and Health Surveys of 2010, 2015 and 2020, we employed the Blinder‐Oaxaca decomposition analysis and the hotspot and cluster analyses to quantify the contributions of key determinants of stunting. Overall, there was a 7.9% and 10.3% points reduction in moderate stunting among urban and rural areas, respectively, and a 2.8% and 8.3% points reduction in severe stunting in urban and rural areas, respectively. Child age, wealth index, maternal education and the number of antenatal care visits were key determinants for the reduction of moderate and severe stunting. Over time, persistent statistically significant hotspots for moderate and severe stunting were observed in Northern and Western parts of the country. There is a need for an adaptive scaling approach when implementing national nutritional interventions by targeting high‐burden regions. Stunting hotspots in Western and Northern provinces underscore the need for coordinated subnational initiatives and strategies such as empowering the rural poor, enhancing antenatal health care, and improving maternal health and education levels to sustain the gains made in reducing childhood stunting.

## Key messages

- Stunting among children under 5 is a major problem in Rwanda, increasing the need for more tailored interventions and increased investments in child health.
- The prevalence of moderate and severe stunting among children aged 0–5 years in Rwanda has reduced over the last decade.
- High hotspots indicating childhood moderate and severe stunting clustered in the Northern and Western provinces of the country remain persistent.
- There is a need for multisectoral collaboration in addressing the key determinants of stunting and sustaining the reduction that has been achieved in the last decade.

## Graphical abstract

Burden of stunting among children under five in Rwanda.

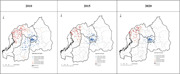

## 1 INTRODUCTION

Stunting is a major public health problem that has short and long‐term health and economic impacts in many developing countries (Chakravarty et al., 2019; De Lucia Rolfe et al., 2018). In 2020, UNICEF, WHO and the World Bank jointly estimated about 144 million children below the age of 5 to be stunted, and of this, 57.5% were from Africa (UNICEF WHO & WB, 2020). Despite extensive investment and comprehensive strategies to address this, the reduction in the prevalence of undernutrition in several sub‐Saharan African (SSA) countries has remained low or insignificant (Stevens et al., 2012) suggesting the need to develop sustainable interventions for underlying determinants of stunting.

Addressing stunting in its moderate and severe forms has been a key policy objective in several SSA countries where its prevalence is higher than the global average (UNICEF WHO & WB, 2020). Rwanda has over the last decade made great investments in the effort to reduce child stunting; from 44% in 2010% to 33% in 2020 while severe stunting reduced from 17% to 9% (NISR et al., 2012; NISR et al., 2021). Furthermore, a rise in the uptake of child health programmes such as vaccination coverage from 90% in 2010 to 96% in 2020, vitamin A supplement administration from 86.4% in 2015 to 86.5% in 2020, children dewormed for helminths from 80.1% in 2015 to 81.5% in 2020 (NISR et al., 2016, 2021) has been observed. Also, governmental and nongovernmental organizations’ driven nutrition improvement strategies such as the *One cow per poor family* Program (*Girinka*) (MINAGRI, 2006), the childhood development program (NECDP, 2017), the Food and Nutrition Policy of 2014 (GRN, 2014), and public health care service delivery (Binagwaho et al., 2020; Nshimyiryo et al., 2019) have been made as a commitment to meet SDGs 2 target 2.2. Concerningly, there are stunting prevalence disparities at subregional levels increasing the need to understand the effect of geographical location on stunting to enhance its reduction through targeted interventions.

Earlier studies based on applying logistic modelling methods have examined the correlates of stunting in Rwanda (Binagwaho et al., 2020; Habimana & Biracyaza, 2019; Nshimyiryo et al., 2019; Uwiringiyimana et al., 2019). Nonetheless, these studies presented limited data for programmatic coverage and geographic setting requirements that would guide policymakers in designing cost‐effective strategies directed at addressing factors with the greatest impact on stunting reduction. Rwanda's population remains largely rural (NISR & MINECOFIN, 2012). Therefore, generating evidence regarding the drivers of stunting reduction for urban and rural areas is key in designing context‐specific nutrition strategies and interventions to optimize program success. Accordingly, we have examined the spatial distribution and determinants of stunting in Rwanda to provide evidence of changes in urban and rural areas for decision‐makers to design area‐specific strategies.

## 2 METHODS

### 2.1 Setting

**Figure 1:**
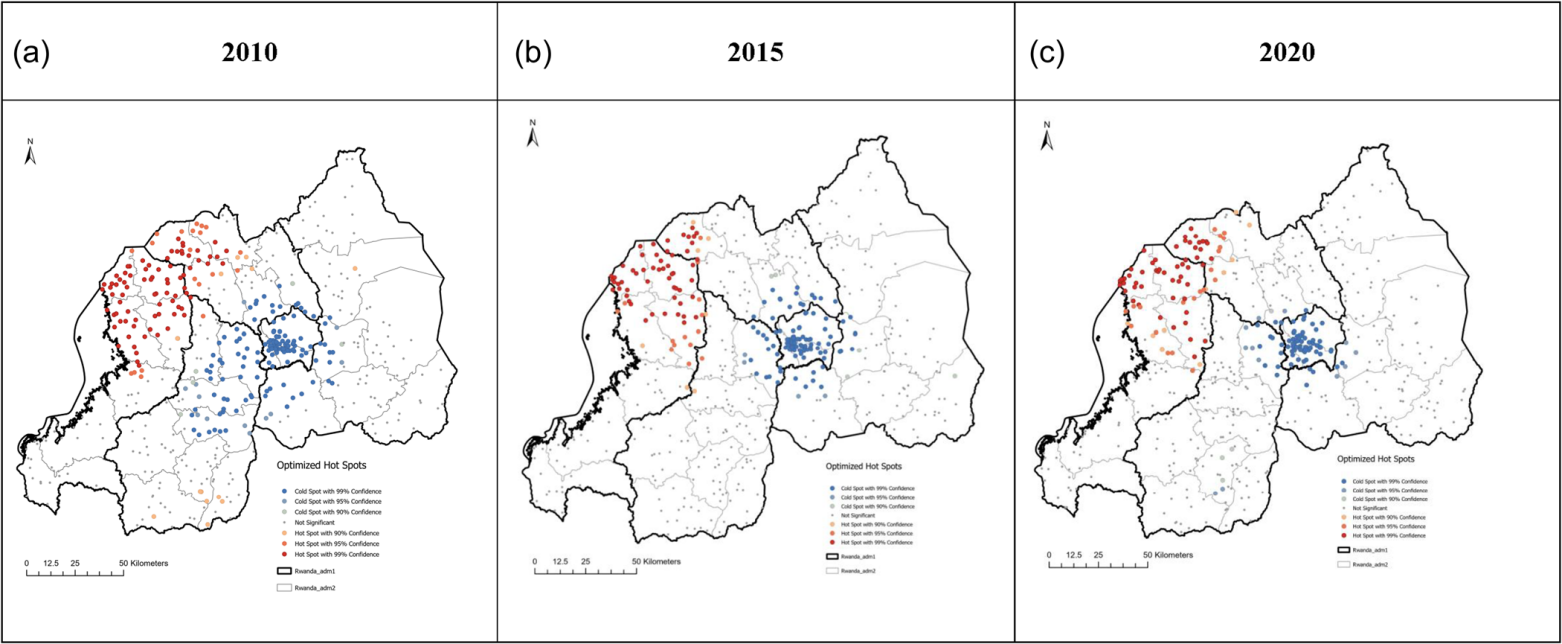
Hotspot and coldspot analysis of moderate stunting clusters: each point data on the map represents a single enumeration area with moderately stunted children for the period (a) 2010, (b) 2015, (c) 2020. Statistically significant high‐risk (hotspot) clusters and low‐risk (cold spot) clusters of moderate stunting. Colours indicate different types of clusters: brown colour‐hotspot clusters of health facilities with higher risk surrounded by areas of relatively lower risk; light to blue—cold spot clusters of lower risk than the surrounding areas. Shades of each colour represent the corresponding significance of results (i.e., white—nonsignificant); progressively darker depending on their probability values.

**Figure 2:**
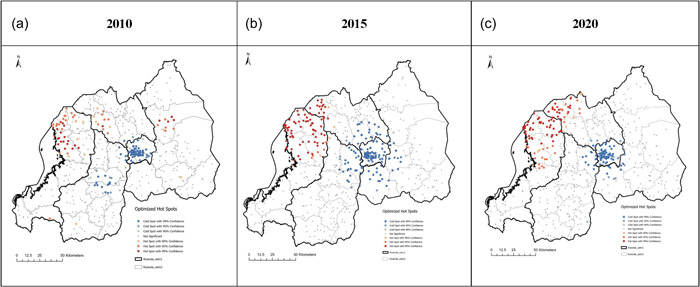
Statistically significant high‐risk (hotspot) clusters and low‐risk (cold spot) clusters of severe stunting for the period (a) 2010, (b) 2015, (c) 2019/20.

The study was conducted in Rwanda, a low‐income and landlocked country in East Africa surrounded by Uganda in the North, Tanzania in the East, the Democratic Republic of Congo in the West, and Burundi in the South. Rwanda has four geopolitical provinces and the City of Kigali (Supporting Information: S1 Figure 1 and S1 Figure 2). With a population size of about 12,955,736 (NISR, 2022) occupying an area of 26,340 km^2^, Rwanda is densely populated and with most people residing in rural areas (NISR & MINECOFIN, 2012). Agriculture is the main livelihood for most households, contributing about 31% to the country's Gross Domestic Product (GDP) and employing about 70% of the population (RDB, 2022). Furthermore, the Agricultural Household Survey (AHS) report of 2020 indicated that there were about 2.3 million agricultural households; for which, agriculture is the main livelihood activity for about 86.3% of the households (NISR, 2021).

### 2.2 Data source and structure

The current study used secondary data from three consecutive Rwanda Demographic and Health Surveys (RDHS) conducted in 2010, 2015 and 2019/2020. The RDHS data is publicly accessible from the Demographic and Health Surveys website (https://dhsprogram.com/) and was accessed after approval from DHS and the University of Global Health Equity (UGHE) Institutional Review Board (Ref: UGHE‐IRB/2022/034). The DHS presents data in the following formats: women's data, children's data, birth data, men's data, and household data. The current study merged the data extracted from the children's recode data, women's, and household data to include health indices for children under the age of 5.

### 2.3 Sample selection

The RDHS data is a nationally representative household survey conducted every 5 years. Respondents included in the RDHS were selected using a multi‐stage, stratified cluster sampling. In this sampling strategy, enumeration areas are the Primary Sampling Units (PSU) while households are sampled at the last stage. To obtain a representative sample, enumeration areas from each province comprise the sampling frame. Using multi‐stage, stratified cluster sampling, the first level of sampling involved cluster selection, which was done using probability proportional to the size, and the second stage involved a systematic sampling of households from the clusters that had been selected. Because of the differences in the geographical structures across the different countries that conduct DHS, modifications in the sampling methodologies may be made to account for country‐specific structures. For Rwanda, the sampling methodologies and reports of findings are available at dhsprogram. com (DHS, 2022). In the current study, the analysis was focused on children under 5 years old. Our sample sizes from the three RDHS were 4010 children in 2010 (4031 weighted cases), 3450 in 2015 (3481 weighted cases) and 3674 in 2020 (3761 weighted cases).

### 2.4 Dependent variables

The main dependent variable was stunting; stratified as moderate and severe. Moderate stunting was defined as having the height‐for‐age *Z*‐score between −3 and −2 standard deviations (−3 SD to −2 SD) from the median of the WHO child growth standards while severe stunting as having the height‐for‐age Z‐score below −3 standard deviations (−3 SD) from the median WHO child growth standards (WHO, 2006, 2018). Anthropometric measurements were taken using standard procedures, while the *z*‐scores were generated by using the WHO‐approved methodologies, thus classifying children as being moderately or severely stunted.

### 2.5 Stratifying variable

The place of residence of the children coded as urban or rural, was used as the stratifying variable. The place of residence for the mother at the time of the survey was taken as the residence of the child.

### 2.6 Covariates

The covariates that were used in this study were extracted based on previously identified factors associated with stunting in Rwanda and elsewhere within SSA (Ali et al., 2017; Binagwaho et al., 2020; Chirande et al., 2015; Habimana & Biracyaza, 2019; Nshimyiryo et al., 2019; Uwiringiyimana et al., 2019). The included covariates were categorized into three levels: child, maternal and household.

Child level covariates included were (0 – 5, 6 –23, 24 –59 months), gender (boy or girl), size at birth (very small, small, average or larger), whether the child had diarrhoea recently, exclusively breastfed, child anaemia, vitamin A supplementation in last 6 months (yes or no), the number of under‐5 children in the family (1, 2, 3, 4+), birth interval (>24 months, 24–47, 48 months+) and consumption of iron‐rich diet (Yes or No), minimum meal frequency, minimum acceptable diet, and minimum dietary diversity. Maternal covariates included, working status (employed or not employed), maternal anaemia status (Yes or No), education level (no education, primary, secondary or higher) and the number of antenatal care visits (0, 1–3, 4+). Household covariates included the household wealth index.

### 2.7 Data analysis

Data analysis was performed using STATA version 17 (StataCorp) (StataCorp, 2021). The STATA survey (svy) command was used to control for the clustering effect. The socio‐demographic characteristics of the participants were analyzed using descriptive statistics and summarized as weighted frequencies and percentages. Pearson's *Χ* ^2^ test was performed to assess the association between the dependent variables (moderate stunting [Yes/No] and severe stunting [Yes/No]) and the covariates and bivariate analysis were used to determine the significance of the association.

The spatial heterogeneity of significant high prevalence areas of moderate and severe stunting was computed for each cluster using the Getis‐Ord G* statistic tools in ESRI's ArcGIS Pro version 2.9. The Getis‐Ord G* statistic was used to classify the autocorrelations into positive and negative correlations. If prevalence rates had similarly high values or low values, they were defined as positive autocorrelation hotspots (represented as High‐High or Low‐Low autocorrelation). If the attributes held opposing high and low values, they were considered to have a negative autocorrelation (representing as High‐Low or Low‐High autocorrelation). To determine the significance of these statistics, *z*‐scores and *p* values at 99%, 95% and 90% confidence levels were used.

To evaluate the observed differences in the outcome variable(moderate stunting [Yes/No] and severe stunting [Yes/No]), we used the Blinder–Oaxaca decomposition technique. Originally designed for use in decomposing labour market outcomes between different groups (Blinder, 1973; Oaxaca, 1973), the technique has become useful in evaluating health outcomes and stratifying the outcomes by socioeconomic class (Geruso, 2012). This technique has also been applied in evaluating child malnutrition across different groups (Uthman, 2009). The current study used the Blinder‐Oaxaca decomposition technique to decompose the difference in the dependent variables (moderate stunting [Yes/No] and severe stunting [Yes/No]) for the period 2010–2020 between rural and urban areas of Rwanda. This is because the difference in the observed moderate and severe stunting levels can be due to the distribution of the covariates of stunting (*compositional differences*), the effects of these covariates on urban and rural communities, or both. In the current study, we used the socioeconomic and demographic covariates classified under child, maternal, and household levels to explain the disparities in child stunting between the rural and urban communities of Rwanda.

## 3 RESULTS

### 3.1 Sociodemographic characteristics of participants

Table 1 shows the characteristics of study participants from the RDHS in 2010, 2015, and 2020. In 2010, 50.8% of children from rural areas were male and this figure reduced to 50% in 2015 before rising to 50.8% in 2020. The proportion of children whose size at birth was very small in urban areas increased from 1.2% in 2010 to 2.2% in 2020. On the other hand, the proportion of children whose size at birth was very small in rural areas reduced from 2.3% in 2010 to 1.8% in 2015, then increased to 2.2% in 2020. The proportion of urban households whose wealth index was poor reduced from 17.6% in 2010 to 10.2% in 2020, while an increase in the proportion of poor households in rural areas was observed between 2010 and 2015 but reduced from 52.7% in 2015 to 49.6% in 2020. In both urban and rural areas, the proportion of mothers with no education reduced between 2010 and 2020, although the number of those with no education in rural areas remained high. Between 2010 and 2015, there was a reduction in the number of children who had been given vitamin A in both urban and rural areas, while marginal rises in urban areas were observed in 2020. There was a reduction in the prevalence of diarrhoea in urban areas while in rural areas, an increase from 12.8% in 2015 to 15.1% in 2020 was observed (Table 1).

**Table 1:** Percentage distribution of background characteristics of children (0–59 months) and mothers, 2010–2020 RDHS.

Background Characteristics	2010 DHS		2015 DHS		2020 DHS	
	Urban %	Rural %	Urban %	Rural %	Urban %	Rural%
*Child's age in months*						
0–5	9.7	8.7	11.2	9.3	9.3	10.3
6–23	28.5	29.5	34.1	32.3	30.2	31.8
24–59	61.8	61.8	54.7	58.4	60.6	57.8
*Sex of child*						
Male	53.2	50.8	50.9	50	48.0	50.8
Female	46.8	49.2	49.1	50	52.0	49.2
*Region*						
Kigali	56.9	2.6	49.3	3.5	54.2	4.5
West	8.4	26.9	17.4	24.0	16.2	23.8
North	7.4	18.4	10.1	17.6	8.2	16.7
South	20.6	24.9	13.1	26.5	9.8	24.7
East	6.7	27.2	10.2	28.4	11.6	30.2
*Child's size at birth*						
Very small	1.2	2.3	1.9	1.8	2.2	2.2
Small	13.8	12.9	11.1	12.9	14.3	15.7
Average or larger	85.0	84.9	86.9	84.3	83.5	82.2
*Wealth index*						
Poor	17.6	48.5	11	52.7	10.2	49.6
Middle	16.4	41.5	16.7	41.5	31.1	40.5
Rich	66.0	10.1	72.2	5.9	58.7	10.0
*Mother's education*						
None	8.2	20.5	7.4	16.3	4.8	**13.1**
Primary	61.5	73.6	57.4	75.6	42.5	69.5
Secondary and higher	30.4	5.9	35.2	8.1	52.7	17.4
*Mother's working status*						
Not working	33.1	18.6	28.8	11.5	38.4	22.4
Working	66.9	81.4	71.2	88.5	61.6	77.6
*Birth order*						
1st	32.6	22.9	32.9	27.1	29.2	23.3
2nd	23.5	18.8	26.5	22.6	26.2	22.0
3rd	15.1	15.0	16.1	15.7	19.0	18.2
4th	10.9	12.4	9	11.7	11.0	13.6
5th+	17.9	30.9	15.5	22.9	14.6	22.9
*Birth interval*						
Less than 24 months	29.3	22.2	20.4	16.4	21.0	16.1
24–47 months	44.7	58.8	44.9	49.8	39.1	46.3
48+ months	26.0	19.0	34.7	33.8	39.9	37.6
*Number of under 5 Children*						
One	44.0	36.0	45.5	44.9	49.0	44.4
Two	43.2	50.5	39.7	43.8	38.1	46.0
Three and above	12.8	13.5	14.8	11.4	12.9	9.7
*Maternal anaemia*						
No	86.5	81.9	85.2	80.6	90.3	87.1
Yes	13.5	18.1	14.8	19.4	9.7	12.9
*Number of ANC visits*						
0	1.4	1.8	1	0.7	1.9	2.2
1–3	57.4	63.2	54.5	55.2	48.6	50.7
4+	41.2	35.0	44.5	44.1	49.5	47.2
*Vitamin A in the last 6 months*						
No	9.2	12.5	22.7	19	18.6	20.2
Yes	90.8	87.5	77.3	81	81.4	79.8
*Child anaemia*						
No	62.7	61.2	69.9	62.1	66.3	62.6
Yes	37.3	38.8	30.1	37.9	33.7	37.4
*Minimum meal frequency*						
No	47.4	50.4	48.7	53.5	47.8	57.4
Yes	52.6	49.6	51.3	46.5	52.2	42.6
*Minimum acceptable diet*						
No	57.4	71.6	65.7	69.7	51.3	61.7
Yes	42.6	28.4	34.3	30.3	48.7	38.3
*Minimum dietary diversity*						
No	86.2	92.6	82.3	90.8	82.3	88.3
Yes	13.8	7.4	17.7	9.2	17.7	11.7
*Consumption of iron‐rich foods*						
No	62.7	81.9	72.5	81.8	62.0	79.4
Yes	37.3	18.1	27.5	18.2	38.0	20.6
*Exclusively breastfed*						
No	98.0	96.8	98.1	97.4	99.1	98.7
Yes	2.0	3.2	1.9	2.6	0.9	1.3
*Presence of diarrhoea*						
No	85.9	86.6	89.9	87.2	88.3	84.9
Yes, in the last 2 weeks	14.1	13.4	10.1	12.8	11.7	15.1

### 3.2 Factors influencing moderate stunting reduction in Rwanda

The prevalence of moderate stunting in rural areas was 46.1% [95% confidence interval (CI): 44.7–48.4] in 2010 and 35.8% [95% CI: 33.8–37.8] in 2020. On the other hand, in urban areas, it was 27.4% [95% CI: 22.5–32.7] in 2010 and 19.5% [95% CI: 16.5– 23.44] in 2020. The overall points change reduction in moderate stunting among under‐5 children between 2010 and 2020 was 7.9% in urban areas and 10.3% in rural areas. In urban areas, major reductions in moderate stunting were recorded in the Northern (33.5%) and the Western regions (25.3%) while in rural areas, major reductions were observed in the Eastern and Southern regions (14.4% and 10.0%), respectively. The study showed a 10.2% points reduction in moderate stunting among children aged 6–23 months living in urban areas and a 7.9% reduction among those living in rural areas. Furthermore, there was a 10.5% points decrease in moderate stunting among male children in urban areas and a 9.6% reduction among those from rural areas. For female children, 4.4% and 11.3% reductions were recorded in urban and rural areas, respectively. Children whose mothers had no education recorded the largest decrease in moderate stunting in urban areas (16.6%) while in rural areas, children whose mothers had a primary level of education recorded the most decline in stunting (9.4%) between 2010 and 2020. Other improvements in moderate stunting were observed among children who achieved the minimum meal frequency (15.5%) in urban areas and (9.9%) in rural areas and those who consumed iron‐rich foods (14.8%) in urban and (14.5%) in rural areas (Supporting Information: S2 Table 1).

### 3.3 Factors influencing severe stunting reduction in Rwanda

The prevalence of severe stunting in rural areas was 18.1% [95% CI: 16.9−19.7] in 2010 and 9.8% [8.9–11.4] in 2020. On the other hand, in urban areas, it was 7.6% [5.6–10.7] in 2010 and 4.8% [3.4 – 6.9] in 2020. The results of the points difference in the prevalence of severe stunting between urban and rural areas are shown in Supporting Information: S2 Table 2. The overall points change reduction in severe stunting between 2010 and 2020 was 2.8% in urban areas and 8.3% in rural areas. In both urban and rural areas, major reductions in severe stunting were recorded in the Eastern region (urban 11.1% vs. rural 11.8%) and Northern region (urban 10.4% vs. rural 7.2%). The study showed a 6.7% points reduction in severe stunting among children aged 6–23 months living in urban areas and an 8.2% reduction among those living in rural areas. Furthermore, there was a 4.9% points decrease in severe stunting among male children living in urban areas and an 8.8% reduction among those living in rural areas. For female children, 0.3% and 7.8% reductions were recorded in urban and rural areas, respectively. Children whose mothers had no education recorded the largest decrease in severe stunting in urban areas (8.6%) while in rural areas, children whose mothers had a primary level of education recorded the most decline in severe stunting (7.5%) between 2010 and 2020. Other improvements in severe stunting levels were observed among children who received the minimum meal frequency (11.4%) in urban areas and (10.6%) in rural areas and those who consumed iron‐rich foods (4.9%) in urban and (7.4%) in rural areas (Supporting Information: S2 Table 2.).

### 3.4 Hot and cold spot analysis in moderate and severe stunting

The spatial hotspot analysis shows clustering of moderate stunting (Figure 1) in the North‐western and Western regions of Rwanda. Variations in the prevalence of moderate stunting were observed between 2010, 2015, and 2020. Based on the Getis − Ord Gi* statistics, the optimized hotspot analysis shows a drift in the geographical distribution of moderate stunting with specific clusters in 2010, 2015 and 2020 cycles and an overall reduction in stunting. In 2010, high rates of moderate stunting were observed in Rubavu, Nyabihu, Rutsiro and Ngororero districts of the Western province, and Musanze, Burera, and Gakenke districts in the Northern province (Figure 1). In 2015, Rubavu, Nyabihu, Ngororero and Musanze districts had significant clusters of moderate stunting, while isolated clusters were observed in Rutsiro. From 2010 through 2020, distinct areas of relatively low cases (cold spots) were observed in the City of Kigali.

Spatial variations of severe stunting at cluster level were observed in the three cycles (Figure 2). In 2010, the spatial analysis at the cluster level showed statistically significant high hotspots of severe stunting in Rubavu, Nyabihu, Rutsiro and Ngororero districts of the Western province, and Gakenke in the Northern province (Figure 2a). In 2015, Rubavu, Nyabihu and Ngororero districts of the Western province had significant clusters of severe stunting, while isolated clusters were observed in Rutsiro (Figure 2b) and Musanze in the Northern province also had significant severe stunting hotspots (Figure 2b). In 2020, statistically significant high hotspots were observed in Rubavu, Musanze and Nyabihu, while isolated hotspots were observed in Rutsiro, Ngororero and Gakenke (Figure 2c). Throughout the study period, the City of Kigali had distinct cold spots.

### 3.5 Decomposition of moderate stunting in urban and rural areas

The overall contribution of the endowments and coefficients to moderate stunting reduction in Rwanda between 2010 and 2020 are presented in Supporting Information: S3 Table 1. There was 24% of the overall percentage change in moderate stunting attributable to the difference in child‐associated characteristics (endowments) while 76% of changes in moderate stunting were due to coefficients.

**Figure 3:**
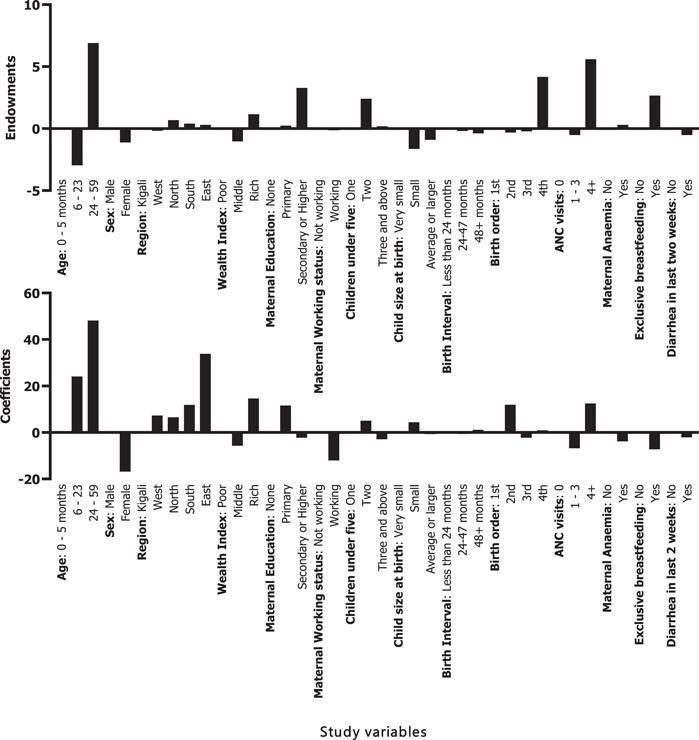
A decomposition analysis of factors contributing to moderate stunting reduction in rural areas.

After controlling for the place of residence, 23.8% of the changes in moderate stunting in rural areas were attributed to changes in child characteristics (endowments), and 76.2% due to coefficients. Major changes in the characteristics of children aged 24–59 months (7%), children from families classified as rich (1.16%), secondary or higher level of education among mothers (3.28%), having more than 4 antenatal care visits (5.59%) and exclusive breastfeeding (2.67%) were observed (Figure 3). In terms of coefficients, changes in the behaviour among mothers with children aged 24‐59 months (48.17%), Southern (12%) and Eastern (34%) provinces, maternal primary education (12%), rich wealth index (14.6%) and more than 4 antenatal care visits (12%) accounted for major contribution in moderate stunting reduction between 2010 and 2020.

**Figure 4:**
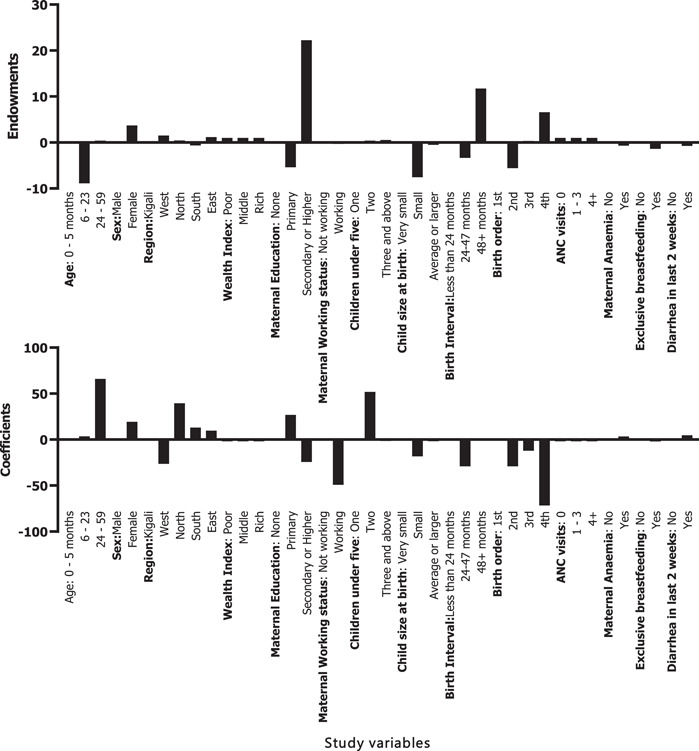
A decomposition analysis of factors contributing to moderate stunting reduction in urban areas.

In urban areas, the estimates of decomposition analysis for the change in moderate stunting showed that endowments were attributed to 23% of the changes, while 77% were attributed to changes in coefficients (Figure 4). In terms of changes attributed to endowments, the mother's education level (secondary or higher: 22%) and birth order of four (6.58%) were the primary contributors explaining the change in the prevalence of moderate stunting between 2010 and 2020. Of the attributable changes in coefficients, mothers with children aged 24–49 months (66.1%), Northern (40%) and Southern (13%) provinces, maternal education (primary: 27%) and having two children below the age of 5 years (52%) explained the change in the prevalence of moderate stunting between 2010 and 2020 (Figure 4).

### 3.6 Decomposition of severe stunting in urban and rural areas

For severe stunting reduction between 2010 and 2020, endowments accounted for 15.2% and coefficients for 87.8% (Supporting Information: S3 Table 2). In rural areas,13.6% of the changes in severe stunting were attributed to child characteristics (endowments) and 86.4% were attributed to coefficients. Major changes in the characteristics of children aged 24–59 months (3%), having more than three children below the age of 5 (1.9%), and birth order (4th) (4.2%). In terms of coefficients, changes in the behaviour among mothers with children aged 24–59 months (40.77%), Eastern province (21%), working mothers (33.78%) and birth order (2nd; 10.1%) accounted for major contributions in severe stunting reduction between 2010 and 2020 (Supporting Information: S4 Table 1).

In urban areas, 84.1% of the changes in severe stunting were attributed to endowments, while 15.9% were attributed to changes in coefficients. In terms of changes attributed to endowments, children from the Eastern province (10.2%), the mother's education level (secondary or higher: 47.22%), and birth order of four (9.3%) having two children below the age of 5 (2.9%) and birth interval of more than 48 months (44.67%) were the primary contributors explaining the change in the prevalence of severe stunting between 2010 and 2020. Of the attributable changes in coefficients, mothers with children aged 6‐23 months (14.2%), children from Eastern province (16.2%), working mothers (18.8%), the birth interval of 24–47 months (42.23%), and maternal anaemia (9%) accounted for most changes in severe stunting reduction in urban areas (Supporting Information: S4 Table 2).

## 4 DISCUSSION

The current study sought to assess the spatial distribution and determinants of moderate and severe stunting reduction among children aged 0–5 years in Rwanda using data from RDHS 2010–2020. This is part of an ongoing effort to generate nuanced evidence under the *Gikuriro Kuri Bose* Program which is an Inclusive Nutrition and Early Child Development (INECD) activity being funded by USAID Rwanda and implemented by Catholic Relief Services (CRS) in close collaboration with eight partners including the University of Global Health Equity (UGHE) and Government of Rwanda to promote nutritional, developmental, health and functional outcomes of children. The results from the study indicate that the prevalence of moderate and severe stunting among children aged 0–5 years in Rwanda has decreased over the last decade. The reduction was more pronounced in rural than urban areas. However, high hotspots indicating childhood moderate and severe stunting clustered in the Northern and Western provinces of the country remain persistent. This outcome suggests the need for increased effort in the implementation of nutrition‐focused interventions. Also, there is a need for future studies to go beyond understanding the role of maternal, child and household characteristics that have been discussed in the current study to other factors such as contextual factors, environmental factors, and socio–cultural practices which are key in influencing childhood stunting.

The current study used the hotspot spatial analysis to identify spatial variation of moderate and severe stunting at the cluster level in Rwanda. This approach also used elsewhere (Haile et al., 2016; Tamir et al., 2022) is essential in identifying specific clusters where childhood stunting remains prevalent and in need of interventions. Despite its persistence, the declining trend of moderate and severe stunting observed in the study can be attributed to strategies such as the implementation of the First Community‐Based Food and Nutrition Programs, the one‐cow‐per‐poor family Programme (*Girinka*) initiative (MINAGRI, 2006), childhood development program (NECDP, 2017) and increased agricultural and livestock production (MINAGRI, 2020). On the other hand, Sekiyama et al. (2020) suggested that the observed persistent moderate and severe stunting in Western and Northern provinces may be due to an over‐reliance on starchy foods with a limited diversity of diet. This suggests the need for nutrition‐specific interventions in combination with health education and the empowerment of women (Vir, 2016).

The results from our study suggest that coefficients (Child factors: child age; household factors: wealth index; Maternal factors: number of antenatal care visits) were the key determinants of moderate and severe stunting reduction in Rwanda. The results from the current study agree with the outcome of studies conducted in Bangladesh and the Philippines which concluded that child‐related factors (age, sex, place of birth and vaccination status), Maternal (education, BMI, stature, media exposure and the number of children), and household (wealth index, family size and sanitation infrastructure) are essential determinants of stunting reduction (Ulep et al., 2021; Win et al., 2021). Furthermore, our study and those of others show that attainment of higher education among mothers increases their chances of access to knowledge of health education, leading to improved child feeding practices. Additionally, educated mothers have improved access to social capital. In our study, data on disease occurrences and sanitation were not available making it difficult to evaluate the effect of these factors on stunting despite having been reported elsewhere as essential determinants of stunting.

Our study observed lower levels of moderate and severe stunting in urban areas than in rural areas. Although stunting reduction was higher in rural areas, the prevalence of stunting remains high in these areas. The disparities observed in our study have best been explained by McDade (2003) who used life history theory and the immune system. According to this author, children from urban areas are more likely to be nutritionally and epidemiologically privileged than those from rural areas. In addition to the availability of various eco‐social amenities in urban areas, children from urban areas are more likely to have better growth due to potentially having a more effective immunity to respond to infections than those in adverse environments such as rural areas. The difference in the growth of children in urban and rural areas has also been documented in Tanzania (Musheiguza et al., 2021), Malawi (Mussa, 2014) and elsewhere (Cardenas et al., 2022; Sharaf & Rashad, 2016). When taken together, our study and those of others suggest that the implementation of stunting reduction strategies should incorporate heterogeneities between urban and rural areas. This is because differences in the levels of determinants would impact the success of the intervention strategies in urban‐rural areas differently. For instance, differences in coefficients would indicate the need for behavioural and awareness programs to be included in intervention strategies while differences in characteristics would indicate the need for more socioeconomic empowerment strategies.

Several studies assessing stunting in Rwanda have been done; however, the current study has applied the decomposition analysis to assess moderate and severe stunting and its spatial variation at the cluster level. By using the geographical approach, our study has highlighted spatial dimensions of understanding moderate and severe stunting in Rwanda. Despite the limitation of the cross‐sectional nature of the data used in this study, which affected the establishment of the causal‐outcome relationship (Sharaf & Rashad, 2016), our findings offer useful insights on the need to design setting tailored policies to enhance the success of stunting reduction programs. Our study further suggests that reducing moderate and severe stunting will require several sectors to act together in designing interventions aimed at improving the socioeconomic circumstances of people and improving health education and nutrition services.

## 5 CONCLUSION

The current study has identified some of the factors that are key in moderate and severe stunting reduction, from the context of the rural and urban setting in Rwanda. Furthermore, the study has identified some of the hotspots of moderate and severe stunting. Overall, these findings suggest a reduction in the prevalence of moderate and severe stunting and the attributable factors influencing this decline. Our results suggest the need for strong and targeted policies specifically to improve the health of children and the socioeconomic status of women especially those from rural areas to bridge the gap between poor and nonpoor. To attain SDG 2, there is a need to redesign nutrition and food security programs in Rwanda to enhance their efficiency and impact. Our study recommends the need for enhanced antenatal check‐ups and mother's education, factors that can help improve child health. Furthermore, there is a need to relook at the regions and clusters with high hotspots of moderate and severe stunting and prioritize them in nutritional interventions. Comprehensive nutritional strategies and health education are needed in these regions to reduce the burden of childhood stunting. To achieve this, there is a need for sustainable multisectoral collaborations between the government and other implementing partners and engaging community‐based organizations that may be key in driving communities to embrace health education and social change.

## AUTHOR CONTRIBUTIONS

Chester Kalinda designed and implemented the study, performed data analysis, and interpretation, developed the first draft, and revised all versions of the manuscript. Million Phiri, Simona J. Simona, Andrew Banda, and Julius Odhiambo Nyerere designed the study and performed data analysis and interpretation of the results. Maria Albin Qambayot, Sage Marie Consolatrice Ishimwe, and Alemayehu Amberbir designed the study and critically reviewed all versions of the manuscript. Rex Wong, Bekele Abebe, and Alemayehu Gebremariam critically reviewed the final manuscript. All authors have read and approved the final manuscript.

## CONFLICT OF INTEREST STATEMENT

The authors declare no conflict of interest.

## Supporting information

Supporting information.

Supporting information.

Supporting information.

Supporting information.

## Data Availability

The raw data supporting the conclusions of this article will be made available by the authors upon request without undue reservation.
