# Supplementary figures and images for: Understanding factors associated with rural‐urban disparities of stunting among under‐five children in Rwanda: A decomposition analysis approach
Source: Matern Child Nutr. 2023 Mar 30;19(3):e13511. doi: 10.1111/mcn.13511 (PMC10262907; doi:10.1111/mcn.13511)

**Supplementary file 1A: Figure 1: Regional administration**


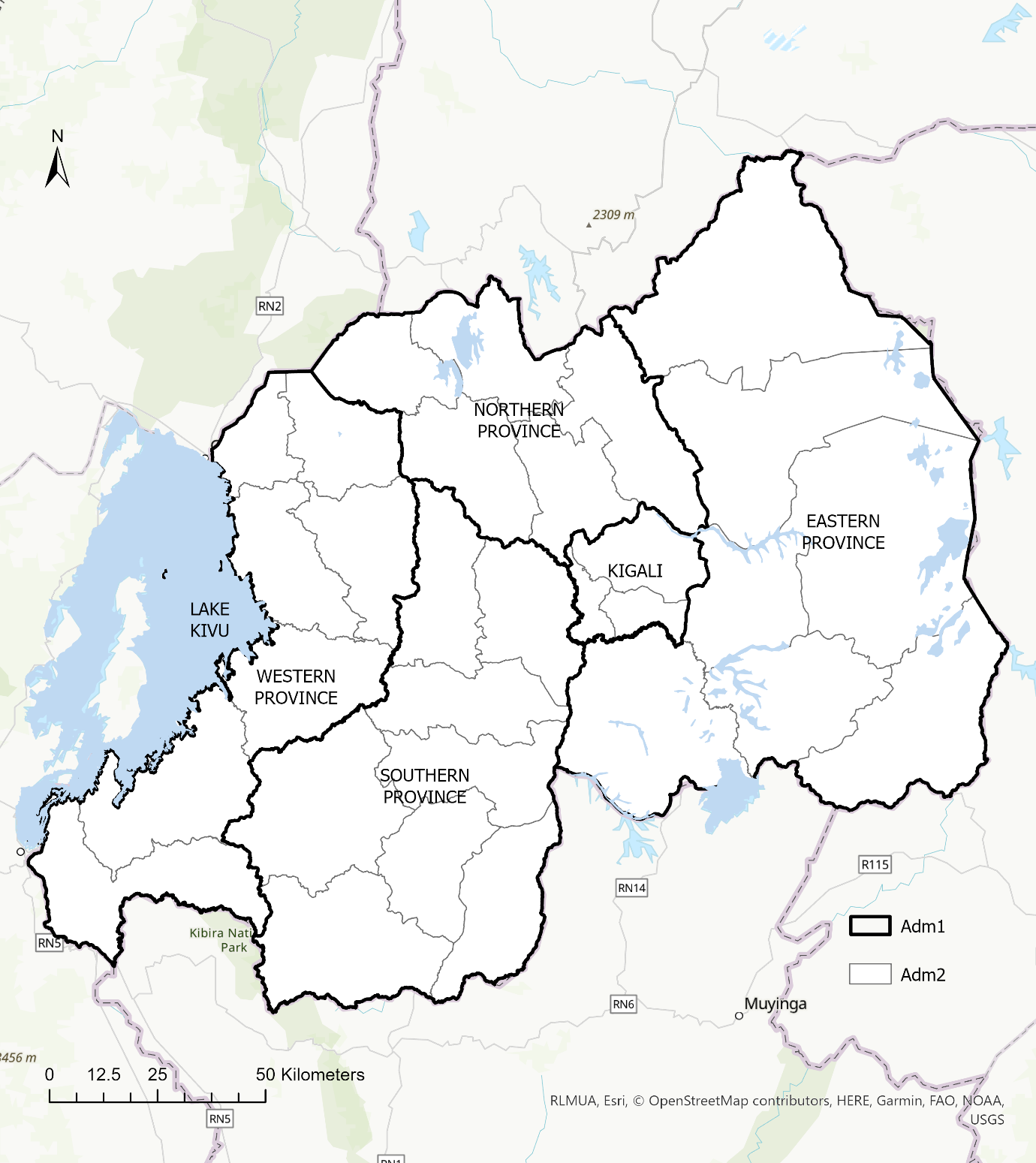


**Supplementary file1: Figure 2: Districts**

**
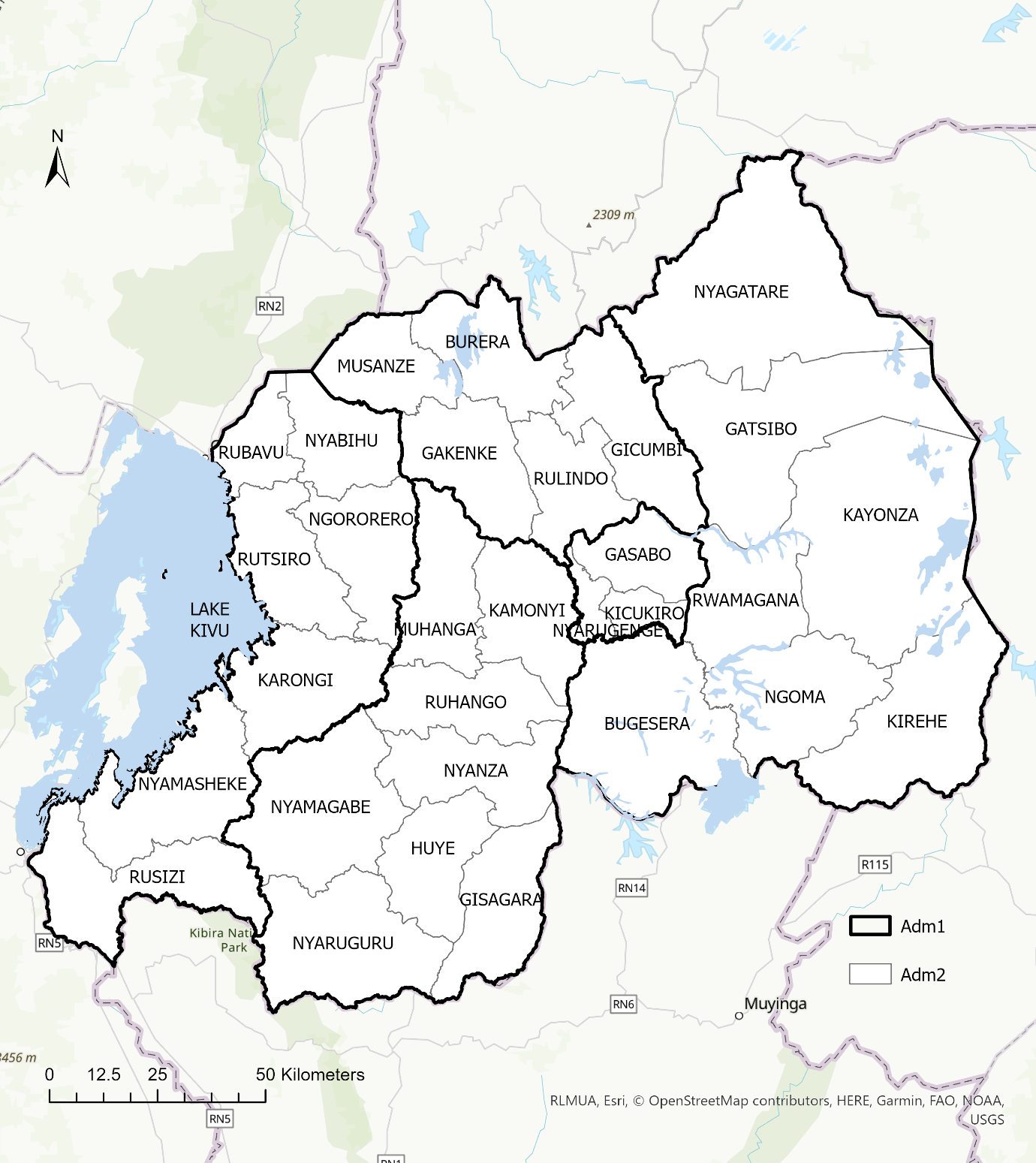
**

Supplement: Supplementary file 1 — Supporting information. [file MCN-19-e13511-s001.docx]
