# Supplementary material for: Understanding factors associated with rural‐urban disparities of stunting among under‐five children in Rwanda: A decomposition analysis approach
Source: Matern Child Nutr. 2023 Mar 30;19(3):e13511. doi: 10.1111/mcn.13511 (PMC10262907; doi:10.1111/mcn.13511)
Supplement: Supplementary file 2 — Supporting information. [file MCN-19-e13511-s004.docx]

**Supplementary file 2: Table 1: Percentage point difference in stunting among children between 2010 - 2020**

| **Background Characteristics** | **2010 DHS (n=4,031)** | | **2015 DHS (n=3,481)** | | **2020 DHS (n=3,761)** | | **Urban Percentage point difference in stunting between 2010 and 2020** | **Rural Percentage point difference in stunting between 2010 and 2020** |
| --- | --- | --- | --- | --- | --- | --- | --- | --- |
|  | Stunting | | Stunting | | Stunting | |  |  |
|  | Urban | Rural | Urban | Rural | Urban | Rural |  |  |
| **Child's Age in months** |  |  |  |  |  |  |  |  |
| **0-5** | 4.3 | 18.4 | 7.1 | 11.5 | 4.4 | 18.1 | **0.1** | **-0.3** |
| **6-23** | 29.3 | 40.9 | 24.5 | 38.8 | 19.1 | 33.0 | **-10.2** | **-7.9** |
| **24-59** | 30.1 | 52.5 | 26.4 | 46.0 | 21.7 | 40.6 | **-8.4** | **-11.9** |
| **Sex of Child** |  |  |  |  |  |  |  |  |
| Male | 32.6 | 49.7 | 28.4 | 45.4 | 22.1 | 40.1 | **-10.5** | **-9.6** |
| Female | 21.5 | 42.6 | 18.4 | 35.6 | 17.1 | 31.3 | **-4.4** | **-11.3** |
| **Region** |  |  |  |  |  |  |  |  |
| Kigali | 21.1 | 30.9 | 20.3 | 28.2 | 18.0 | 27.8 | **-3.1** | **-3.1** |
| West | 49.7 | 49.3 | 31.7 | 46.8 | 24.4 | 42.5 | **-25.3** | **-6.8** |
| North | 62.0 | 49.8 | 25.6 | 40.8 | 28.5 | 41.5 | **-33.5** | **-8.3** |
| South | 22.5 | 43.8 | 22.9 | 42.1 | 19.4 | 33.8 | **-3.1** | **-10.0** |
| East | 31.0 | 44.3 | 24.0 | 35.7 | 15.8 | 29.9 | **-15.2** | **-14.4** |
| **Child's Size at Birth** |  |  |  |  |  |  |  |  |
| Very small | 27.0 | 64.0 | 27.8 | 64.1 | 27.2 | 65.3 | **0.2** | **1.3** |
| Small | 29.9 | 53.4 | 33.4 | 52.8 | 30.8 | 47.9 | **0.9** | **-5.5** |
| Average or larger | 26.7 | 44.6 | 22.5 | 37.9 | 17.3 | 32.8 | **-9.4** | **-11.8** |
| **Wealth index** |  |  |  |  |  |  |  |  |
| Poor | 46.2 | 52.6 | 51.5 | 46.8 | 41.1 | 44.6 | **-5.1** | **-8.0** |
| Middle | 34.4 | 42.3 | 27.7 | 34.3 | 33.3 | 30.4 | **-1.1** | **-11.9** |
| Rich | 20.7 | 30.7 | 18.7 | 25.1 | 8.5 | 13.8 | **-12.2** | **-16.9** |
| **Mother’s Education** |  |  |  |  |  |  |  |  |
| None | 54.7 | 51.4 | 43.2 | 47.8 | 38.1 | 45.6 | **-16.6** | **-5.8** |
| Primary | 31.1 | 45.8 | 28.0 | 40.8 | 25.5 | 36.4 | **-5.6** | **-9.4** |
| Secondary & Higher | 12.4 | 31.0 | 13.0 | 24.8 | 12.1 | 25.7 | **-0.3** | **-5.3** |
| **Mother's Working Status** |  |  |  |  |  |  |  |  |
| Not working | 30.2 | 45.9 | 20.4 | 38.6 | 17.5 | 33.0 | **-12.7** | **-12.9** |
| Working | 26.0 | 46.2 | 24.9 | 40.8 | 20.8 | 36.6 | **-5.2** | **-9.6** |
| **Birth Order** |  |  |  |  |  |  |  |  |
| 1st | 19.6 | 38.9 | 23.5 | 37.4 | 14.2 | 34.3 | **-5.4** | **-4.6** |
| 2nd | 27.0 | 45.5 | 19.0 | 38.4 | 12.7 | 33.0 | **-14.3** | **-12.5** |
| 3rd | 35.0 | 47.9 | 28.7 | 40.6 | 21.5 | 35.5 | **-13.5** | **-12.4** |
| 4^th^ | 32.1 | 46.5 | 35.1 | 43.2 | 20.7 | 35.6 | **-11.4** | **-10.9** |
| 5th + | 33.0 | 50.9 | 20.7 | 45.2 | 35.7 | 40.2 |  |  |
| **Birth Interval** |  |  |  |  |  |  | **0.0** | **0.0** |
| Less than 24 months | 38.5 | 47.9 | 21.2 | 43.9 | 26.7 | 39.0 | **-11.8** | **-8.9** |
| 24-47 months | 30.7 | 49.4 | 26.7 | 42.2 | 21.4 | 37.3 | **-9.3** | **-12.1** |
| 48+ months | 23.9 | 45.1 | 21.6 | 39.9 | 19.5 | 33.5 | **-4.4** | **-11.6** |
| **Number of Under 5 Children** |  |  |  |  |  |  |  |  |
| One | 22.7 | 43.4 | 22.5 | 38.9 | 17.3 | 33.0 | **-5.4** | **-10.4** |
| Two | 32.9 | 48.6 | 24.2 | 43.1 | 20.1 | 37.5 | **-12.8** | **-11.1** |
| Three and above | 26.7 | 44.5 | 26.2 | 36.7 | 27.6 | 39.6 | **-0.9** | **-4.9** |
| **Maternal Anaemia** |  |  |  |  |  |  |  |  |
| No | 28.2 | 46.7 | 23.5 | 40.4 | 19.5 | 35.3 | **-8.7** | **-11.4** |
| Yes | 24.9 | 43.7 | 23.6 | 41.1 | 19.2 | 38.6 | **-5.7** | **-5.1** |
| **Number of ANC visits** |  |  |  |  |  |  |  |  |
| 0 | 20.4 | 46.9 | 28.6 | 51.7 | 60.5 | 39.3 | **40.1** | **-7.6** |
| 1 – 3 | 30.1 | 43.6 | 24.5 | 40.8 | 19.9 | 37.1 | **-10.2** | **-6.5** |
| 4+ | 19.9 | 41.9 | 21.8 | 35.2 | 14.5 | 29.3 | **5.4** | **-12.6** |
| **Vitamin A in last 6 months** |  |  |  |  |  |  |  |  |
| No | 10.9 | 27.9 | 16.2 | 27.2 | 9.1 | 21.4 | **-1.8** | **-6.5** |
| Yes | 29.2 | 48.7 | 25.8 | 43.5 | 21.7 | 37.3 | **-7.5** | **-11.4** |
| **Child Anaemia** |  |  |  |  |  |  |  |  |
| No | 29.0 | 49.5 | 23.2 | 42.5 | 21.0 | 36.4 | **-8.0** | **-13.1** |
| Yes | 32.5 | 48.1 | 32.2 | 45.4 | 20.4 | 40.4 | **-12.1** | **-7.7** |
| **Minimum Meal Frequency** |  |  |  |  |  |  |  |  |
| **No** | 26.2 | 41.3 | 29.5 | 36.9 | 21.8 | 34.9 | **-4.4** | **-6.4** |
| **Yes** | 32.0 | 40.4 | 19.6 | 41.0 | 16.5 | 30.5 | **-15.5** | **-9.9** |
| **Minimum Acceptable Diet** |  |  |  |  |  |  |  |  |
| **No** | 29.5 | 45.2 | 24.9 | 40.1 | 21.2 | 35.4 | **-8.3** | **-9.8** |
| **Yes** | 24.7 | 48.1 | 21.4 | 41.2 | 17.7 | 36.4 | **-7.0** | **-11.7** |
| **Minimum Dietary Diversity** |  |  |  |  |  |  |  |  |
| **No** | 30.9 | 49.4 | 26.8 | 44.4 | 21.6 | 38.9 | **-9.3** | **-10.5** |
| **Yes** | 23.5 | 41.1 | 20.3 | 33.3 | 17.1 | 30.1 | **-6.4** | **-11.0** |
| **Consumption of Iron-Rich Foods** |  |  |  |  |  |  |  |  |
| **No** | 28.2 | 40.8 | 25.3 | 39.1 | 20.8 | 34.5 | **-7.4** | **-6.3** |
| **Yes** | 31.0 | 41.1 | 22.4 | 37.5 | 16.2 | 26.6 | **-14.8** | **-14.5** |
| **Exclusively breastfed** |  |  |  |  |  |  |  |  |
| **No** | 25.6 | 44.8 | 22.6 | 39.1 | 19.4 | 34.9 | **-6.2** | **-9.9** |
| **Yes** | 36.7 | 53.5 | 33.9 | 49.5 | 24.9 | 45.0 | **-11.8** | **-8.5** |
| **Presence of Diarrhea** |  |  |  |  |  |  |  |  |
| **No** | 25.6 | 46.6 | 21.9 | 39.6 | 18.1 | 34.7 | **-7.5** | **-11.9** |
| **Yes, in last 2 weeks** | 38.9 | 43.1 | 36.5 | 46.4 | 28.5 | 41.3 | **-10.4** | **-1.8** |
| **Total** | 27.4 | 46.1 | 23.6 | 40.5 | 19.5 | 35.8 | **-7.9** | **-10.3** |

**Supplementary file 2: Table 2:** Percentage point difference in severe stunting among children between 2010 - 2020

| **Background Characteristics** | **2010 DHS (n=4,031)** | | **2015 DHS (n=3,481)** | | **2020 DHS (n=3,761)** | | **Urban Percentage point difference in severe stunting between 2010 and 2020** | **Rural Percentage point difference in severe stunting between 2010 and 2020** |
| --- | --- | --- | --- | --- | --- | --- | --- | --- |
|  | Severe  Stunting | | Severe Stunting | | Severe  Stunting | |  |  |
|  | Urban | Rural | Urban | Rural | Urban | Rural |  |  |
| **Child's Age in months** |  |  |  |  |  |  |  |  |
| **0-5** | 0.0 | 5.0 | 4.1 | 3.7 | 0.0 | 5.2 | **0.0** | **0.2** |
| **6-23** | 12.2 | 16.4 | 9.5 | 13.9 | 5.5 | 8.2 | **-6.7** | **-8.2** |
| **24-59** | 6.7 | 20.7 | 5.5 | 16.2 | 5.1 | 11.5 | **-1.6** | **-9.2** |
| **Sex of Child** |  |  |  |  |  |  |  |  |
| Male | 10.2 | 20.9 | 7.9 | 16.7 | 5.3 | 12.1 | **-4.9** | **-8.8** |
| Female | 4.7 | 15.3 | 5.3 | 11.9 | 4.4 | 7.5 | **-0.3** | **-7.8** |
| **Region** |  |  |  |  |  |  |  |  |
| Kigali | 6.2 | 13.4 | 4.8 | 6.3 | 4.8 | 8.1 | **-1.4** | **-5.3** |
| West | 11.4 | 20.4 | 11.3 | 19.7 | 5.7 | 14.3 | **-5.7** | **-6.1** |
| North | 19.3 | 19.1 | 5.6 | 14.1 | 8.9 | 11.9 | **-10.4** | **-7.2** |
| South | 4.3 | 15.4 | 5.9 | 14.6 | 2.5 | 9.6 | **-1.8** | **-5.8** |
| East | 14.4 | 18.1 | 11.4 | 12.1 | 3.3 | 6.3 | **-11.1** | **-11.8** |
| **Child's Size at Birth** |  |  |  |  |  |  |  |  |
| Very small | 18.6 | 25.6 | 13.0 | 21.5 | 16.6 | 27.8 | **-2.0** | **2.2** |
| Small | 13.1 | 23.0 | 10.3 | 21.4 | 10.1 | 15.9 | **-3.0** | **-7.1** |
| Average or larger | 7.0 | 17.2 | 6.2 | 13.0 | 3.6 | 8.3 | **-3.4** | **-8.9** |
| **Wealth index** |  |  |  |  |  |  |  |  |
| Poor | 15.3 | 22.0 | 19.9 | 17.7 | 13.1 | 13.4 | **-2.2** | **-8.6** |
| Middle | 13.1 | 15.3 | 9.1 | 10.9 | 7.9 | 7.5 | **-5.2** | **-7.8** |
| Rich | 4.3 | 11.0 | 4.2 | 6.5 | 1.7 | 1.7 | **-2.6** | **-9.3** |
| **Mother’s Education** |  |  |  |  |  |  |  |  |
| None | 17.1 | 22.0 | 18.4 | 18.0 | 8.5 | 15.1 | **-8.6** | **-6.9** |
| Primary | 9.0 | 17.5 | 6.9 | 14.2 | 8.3 | 10.0 | **-0.7** | **-7.5** |
| Secondary & higher | 2.3 | 11.1 | 4.3 | 8.2 | 1.3 | 4.9 | **-1.0** | **-6.2** |
| **Mother's Working Status** |  |  |  |  |  |  |  |  |
| Not working | 8.4 | 16.9 | 9.8 | 18.3 | 4.6 | 11.5 | **-3.8** | **-5.4** |
| Working | 7.2 | 18.4 | 5.6 | 13.8 | 5.0 | 9.3 | **-2.2** | **-9.1** |
| **Birth Order** |  |  |  |  |  |  |  |  |
| 1st | 5.1 | 13.5 | 6.2 | 12.9 | 1.8 | 8.2 | **-3.3** | **-5.3** |
| 2nd | 10.5 | 16.7 | 4.7 | 13.0 | 3.0 | 8.8 | **-7.5** | **-7.9** |
| 3rd | 9.0 | 20.5 | 9.6 | 15.0 | 4.2 | 8.0 | **-4.8** | **-12.5** |
| 4th | 5.0 | 20.9 | 10.3 | 14.9 | 4.6 | 11.3 | **-0.4** | **-9.6** |
| 5th + | 9.4 | 20.1 | 6.1 | 16.6 | 14.0 | 13.0 | **4.6** | **-7.1** |
| **Birth Interval** |  |  |  |  |  |  |  |  |
| Less than 24 months | 8.4 | 18.5 | 6.4 | 14.9 | 12.4 | 11.5 | **4.0** | **-7.0** |
| 24-47 months | 10.8 | 20.0 | 7.3 | 15.3 | 5.2 | 11.9 | **-5.6** | **-8.1** |
| 48+ months | 5.7 | 19.1 | 6.6 | 14.0 | 3.7 | 7.7 | **-2.0** | **-11.4** |
| **Number of Under 5 Children** |  |  |  |  |  |  |  |  |
| One | 4.9 | 15.7 | 6.7 | 13.6 | 2.5 | 7.6 | **-2.4** | **-8.1** |
| Two | 11.0 | 19.8 | 6.6 | 15.6 | 6.6 | 10.5 | **-4.4** | **-9.3** |
| Three and above | 7.4 | 18.5 | 7.0 | 12.4 | 9.4 | 16.5 | **2.0** | **-2.0** |
| **Maternal Anaemia** |  |  |  |  |  |  |  |  |
| No | 7.3 | 18.0 | 6.8 | 13.9 | 4.9 | 9.4 | **-2.4** | **-8.6** |
| Yes | 10.2 | 18.7 | 6.1 | 16.1 | 4.3 | 12.2 | **-5.9** | **-6.5** |
| **Number of ANC visits** |  |  |  |  |  |  |  |  |
| 0 | 7.0 | 11.8 | 25.1 | 14.8 | 27.0 | 10.4 | **20.0** | **-1.4** |
| 1 - 3 | 9.2 | 16.5 | 8.4 | 14.7 | 4.1 | 9.9 | **-5.1** | **-6.6** |
| 4+ | 6.4 | 15.8 | 5.2 | 10.7 | 3.8 | 6.8 | **-2.6** | **-9.0** |
| **Vitamin A in last 6 months** |  |  |  |  |  |  |  |  |
| No | 1.3 | 9.6 | 6.3 | 9.2 | 3.1 | 6.5 | **1.8** | **-3.1** |
| Yes | 8.3 | 19.3 | 6.8 | 15.5 | 5.5 | 10.0 | **-2.8** | **-9.3** |
| **Child Anaemia** |  |  |  |  |  |  |  |  |
| No | 6.5 | 18.4 | 4.7 | 14.1 | 5.2 | 9.4 | **-1.3** | **-9.0** |
| Yes | 12.0 | 21.0 | 12.5 | 17.5 | 5.4 | 12.0 | **-6.6** | **-9.0** |
| **Minimum Meal Frequency** |  |  |  |  |  |  |  |  |
| **No** | 10.4 | 15.9 | 10.6 | 13.3 | 8.5 | 9.6 | **-1.9** | **-6.3** |
| **Yes** | 13.8 | 17.0 | 8.3 | 14.6 | 2.4 | 6.4 | **-11.4** | **-10.6** |
| **Minimum Acceptable Diet** |  |  |  |  |  |  |  |  |
| **No** | 7.7 | 18.9 | 7.7 | 15.1 | 5.3 | 10.0 | **-2.4** | **-8.9** |
| **Yes** | 7.6 | 16.3 | 4.7 | 12.6 | 4.4 | 9.6 | **-3.2** | **-6.7** |
| **Minimum Dietary Diversity** |  |  |  |  |  |  |  |  |
| **No** | 8.2 | 19.4 | 7.4 | 15.8 | 5.4 | 10.7 | **-2.8** | **-8.7** |
| **Yes** | 10.1 | 17.8 | 5.2 | 10.8 | 4.5 | 8.0 | **-5.6** | **-9.8** |
| **Consumption of Iron-Rich Foods** |  |  |  |  |  |  |  |  |
| **No** | 12.2 | 16.9 | 10.8 | 14.3 | 4.5 | 8.5 | **-7.7** | **-8.4** |
| **Yes** | 12.1 | 14.4 | 6.0 | 12.2 | 7.2 | 7.0 | **-4.9** | **-7.4** |
| **Exclusively breastfed** |  |  |  |  |  |  |  |  |
| **No** | 8.1 | 17.5 | 7.0 | 14.1 | 5.1 | 9.7 | **-3.0** | **-7.8** |
| **Yes** | 4.0 | 21.6 | 7.1 | 18.1 | 1.8 | 13.8 | **1.1** | **-7.8** |
| **Presence of Diarrhoea** |  |  |  |  |  |  |  |  |
| **No** | 6.7 | 18.0 | 6.0 | 13.6 | 4.8 | 9.3 | **-1.9** | **-8.7** |
| **Yes, in last 2 weeks** | 13.6 | 18.6 | 11.5 | 19.1 | 5.1 | 12.7 | **-8.5** | **-5.9** |
| **Total** | 7.6 | 18.1 | 6.7 | 14.3 | 4.8 | 9.8 | **-2.8** | **-8.3** |
