# Supplementary material for: Understanding factors associated with rural‐urban disparities of stunting among under‐five children in Rwanda: A decomposition analysis approach
Source: Matern Child Nutr. 2023 Mar 30;19(3):e13511. doi: 10.1111/mcn.13511 (PMC10262907; doi:10.1111/mcn.13511)
Supplement: Supplementary file 3 — Supporting information. [file MCN-19-e13511-s002.docx]

**Supplementary file S4 Table Table1.** Overall contribution of the endowments and coefficients to stunting reduction in Rwanda

| **Background Characteristics** | **Due to differences in characteristics (E)** | | **Due to differences in coefficients (C)** | |
| --- | --- | --- | --- | --- |
|  | **Endowments (95% CI)** | **%** | **Coefficients (95% CI)** | **%** |
| **Child's Age in months** |  |  |  |  |
| **0 - 5** | Ref |  | Ref |  |
| **6 - 23** | 0.00321*** ( 0.00186, 0.00455) | -3.06 | -0.02215 (-0.06516, 0.02086) | 21.14 |
| **24 - 59** | -0.00567*** (-0.00767 , -0.00368) | 5.41 | -0.04533 (-0.11244, 0.02179) | 43.26 |
| **Sex of Child** |  |  |  |  |
| Male | Ref |  | Ref |  |
| Female | 0.00028*** ( 0.00015, 0.00040) | -0.26 | 0.01653 (-0.01179, 0.04486) | -15.78 |
| **Residence** |  |  |  |  |
| Urban | Ref |  | Ref |  |
| Rural | -0.00011 ( -0.00169, 0.00146) | 0.11 | -0.00627 (-0.09901, 0.08648) | 5.98 |
| **Region** |  |  |  |  |
| Kigali | Ref |  | Ref |  |
| West | 0.00089 (-0.00095, 0.00256) | -0.77 | -0.01200 (-0.04394, 0.01993) | 11.46 |
| North | -0.00009 (-0.00025, 0.00007) | 0.09 | -0.01762 (-0.05084, 0.01559) | 16.82 |
| South | -0.00028 (-0.00106, 0.00051) | 0.26 | -0.01721 (-0.04122, 0.00680) | 16.43 |
| East | -0.00086 (-0.00200, 0.00028) | 0.82 | -0.03920* (-0.07375, -0.00464) | 37.41 |
| **Wealth index** |  |  |  |  |
| Poor | Ref |  | Ref |  |
| Middle | 0.00029*** ( 0.00013, 0.00046) | -0.28 | 0.00615 (-0.01837, 0.03068) | -5.87 |
| Rich | -0.00474*** (-0.00200, 0.00028) | 4.53 | -0.02644** (-0.04336, -0.00952) | 25.23 |
| **Mother’s Education** |  |  |  |  |
| None | Ref |  | Ref |  |
| Primary | 0.00025 (-0.00017, 0.00067) | -0.24 | -0.00693 (-0.05802, 0.04417) | 6.61 |
| Secondary or Higher | -0.00371 (-0.00969, 0.00226) | 3.55 | 0.00686 (-0.00342, 0.01713) | -6.54 |
| **Mother's Working Status** |  |  |  |  |
| Not working | Ref |  | Ref |  |
| Working | -0.00032 (-0.00100, 0.00035) | 0.31 | 0.01429 (-0.04327, 0.07185) | -13.64 |
| **Number of children under 5 in Household** |  |  |  |  |
| One | Ref |  | Ref |  |
| Two | -0.00210 (-0.00505, 0.00085) | 2.00 | -0.00743 (-0.04469, 0.02983) | 7.09 |
| Three and above | -0.00005 (-0.00153, 0.00142) | 0.05 | 0.00291 (-0.00991, 0.01572) | -2.78 |
| **Child's Size at Birth** |  |  |  |  |
| Very small | Ref |  | Ref |  |
| Small | 0.00236*** (0.00141, 0.00331) | -2.25 | -0.00068 (-0.01002, 0.00866) | 0.65 |
| Average or larger | 0.00082*** (0.00037, 0.00126) | -0.78 | 0.00011 (-0.00340, 0.00363) | -0.11 |
| **Birth Interval** |  |  |  |  |
| Less than 24 months | Ref |  | Ref |  |
| 24-47 months | 0.00123 (-0.00400, 0.00646) | -1.17 | -0.00027 (-0.04448, 0.04395) | 0.25 |
| 48+ months | -0.00123 (-0.01180, 0.00934) | 1.17 | 0.00089 (-0.01892, 0.02069) | -0.85 |
| **Birth order** |  |  |  |  |
| 1st | Ref |  | Ref |  |
| 2nd | 0.00109 (-0.00106, 0.00324) | -1.04 | -0.00761 (-0.02364, 0.00841) | 7.26 |
| 3rd | 0.00022 (-0.00008, 0.00052) | -0.21 | 0.0039 (-0.00986, 0.01765) | -3.72 |
| 4th | -0.00588* (-0.01066, -0.00110) | 5.61 | 0.00812 (-0.02225, 0.03849) | -7.75 |
| **Number of ANC visits** |  |  |  |  |
| 0 | Ref |  | Ref |  |
| 1 - 3 | 0.00728 (-0.00352, 0.01807) | -6.95 | -0.05600 (-0.18204, 0.07004 ) | 53.45 |
| 4+ | -0.01267* (-0.02479, -0.)00055 | 12.1 | -0.04037 (-0.10465, 0.02390) | 38.53 |
| **Maternal Anaemia** |  |  |  |  |
| No | Ref |  | Ref |  |
| Yes | 0.00004 (-0.00220, 0.00228) | -0.04 | 0.00013 (-0.01384, 0.01411) | -0.13 |
| **Exclusively breastfed** |  |  |  |  |
| No | Ref |  | Ref |  |
| Yes | -0.00228 (-0.00774, 0.00319) | 2.17 | 0.00499 (-0.01458, 0.02455) | -4.76 |
| **Presence of Diarrhea** |  |  |  |  |
| No | Ref |  | Ref |  |
| Yes, in last 2 weeks | 0.00058 (-0.00071, 0.00187) | -0.56 | 0.00219 (-0.00852, 0.01289) | -2.09 |
| **Total** | **-0.02519** (-0.04133, -0.00905)** | **24.04** | **-0.07958*** (-0.11137, -0.04780)** | **75.96** |

**Supplementary file S4 Table 2. Overall contribution of the endowments and coefficients to severe stunting reduction in Rwanda**

| **Background Characteristics** | **Due to differences in characteristics (E)** | | **Due to differences in coefficients (C)** | |
| --- | --- | --- | --- | --- |
|  | **Endorments** | **%** | **Coefficients** | **%** |
| **Child's Age in months** |  |  |  |  |
| **0 - 5** | Ref |  | Ref |  |
| **6 - 23** | 0.0012* (0.00006, 0.00234) | -1.43 | -0.02020 (-0.04681 , 0.00641) | 24.2 |
| **24 - 59** | -0.00197** (-0.00319, -0.00075) | 2.36 | -0.03356 (-0.09171 , 0.02459) | 40.2 |
| **Sex of Child** |  |  |  |  |
| Male | Ref |  | Ref |  |
| Female | 0.00016** (0.00006, 0.00026) | -0.19 | 0.00703 ( -0.00990, 0.02396) | -8.42 |
| **Residence** |  |  |  |  |
| Urban | Ref |  | Ref |  |
| Rural | -0.00012 (-0.00080 , 0.00055) | 0.15 | -0.030`9 (-0.09201 , 0.03162) | 36.17 |
| **Region** |  |  |  |  |
| Kigali | Ref |  | Ref |  |
| West | 0.000978* (0.00010 , 0.00183) | -1.16 | -0.00473 (-0.02465 , 0.01518) | 5.67 |
| North | -0.00002 (-0.00014, 0.00010) | 0.02 | -0.00909 (-0.02959, 0.01141) | 10.89 |
| South | 0.00003 (-0.00017, 0.00023) | -0.04 | -0.00603 (-0.01998, 0.00792) | 7.23 |
| East | -0.00043** (-0.00073, -0.00014) | 0.52 | -0.02365* (-0.04510, -0.00221 ) | 28.34 |
| **Wealth index** |  |  |  |  |
| Poor | Ref |  | Ref |  |
| Middle | 0.00023* ( 0.00006 , 0.00039) | -0.27 | 0.00364 (-0.01133, 0.01860) | -4.36 |
| Rich | -0.00187*** ((-0.00281 , -0.00092) | 2.24 | -0.01391* (-0.02644, -0.00138) | 16.66 |
| **Mother’s Education** |  |  |  |  |
| None | Ref |  | Ref |  |
| Primary | 0.00011 (-0.00008, 0.00029) | -0.13 | 0.00097 (-0.02730 , 0.02924) | -1.16 |
| Secondary or Higher | -0.00259 (-0.00558, 0.00039) | 3.11 | 0.00099 (-0.00554, 0.00751) | -1.18 |
| **Mother's Working Status** |  |  |  |  |
| Not working | Ref |  | Ref |  |
| Working | 0.00012 (-0.00022 , 0.00047) | -0.15 | -0.02446 (-0.05809 , 0.00918) | 29.3 |
| **Number of children under 5 in Household** |  |  |  |  |
| One | Ref |  | Ref |  |
| Two | -0.00098* (-0.00185, -0.00012) | 1.18 | 0.00404 (-0.01867, 0.02675) | -4.84 |
| Three and above | -0.00124** (-0.00203, -0.00044) | 1.48 | 0.01047* ( 0.00145 , 0.01948) | -12.54 |
| **Child's Size at Birth** |  |  |  |  |
| Very small | Ref |  | Ref |  |
| Small | 0.00064*** (0.00030 , 0.00098) | -0.77 | 0.00320 (-0.00211, 0.00851) | -3.83 |
| Average or larger | 0.00007*** (0.00003, 0.00011) | -0.08 | 0.00220* (0.00042, 0.00398 ) | -2.63 |
| **Birth Interval** |  |  |  |  |
| Less than 24 months | Ref |  | Ref |  |
| 24-47 months | 0.00014 (-0.00167, 0.00194) | -0.16 | -0.00210 (-0.01705, 0.00289) | 2.51 |
| 48+ months | -0.00135 (-0.00539 , 0.00270) | 1.61 | -0.00503 (-0.00757, 0.00867) | 6.02 |
| **Birth order** |  |  |  |  |
| 1st | Ref |  | Ref |  |
| 2nd | -0.00029 (-0.00124, 0.00067) | 0.34 | -0.00708 (-0.01705, 0.00289) | 8.48 |
| 3rd | 0.00012 (-0.00011, 0.00036) | -0.15 | 0.00055 (-0.0075, 0.00867) | -0.66 |
| 4th | -0.00149 (-0.00322, 0.00023 ) | 1.79 | 0.00586 (-0.01184, 0.02355 ) | -7.02 |
| **Maternal Anaemia** |  |  |  |  |
| No | Ref |  | Ref |  |
| Yes | -0.00025 (-0.00114, 0.00064) | 0.3 | -0.00095 (-0.00921, 0.00730) | 1.14 |
| **Exclusively breastfed** |  |  |  |  |
| No | Ref |  | Ref |  |
| Yes | -0.00081 (-0.00256, 0.00095) | 0.97 | 0.00478 ( -0.00434, 0.01391) | -6.73 |
| **Presence of Diarrhea** |  |  |  |  |
| No | Ref |  | Ref |  |
| Yes, in last 2 weeks | 0.00015 (-0.00042, 0.00071 ) | -0.17 | -0.00117 (-0.00696, 0.00462 ) | 1.4 |
| **Total** | **-0.01272** (-0.02113, -0.00430)** | **15.23** | **-0.07076*** (-0.09083, -0.05069)** | **84.77** |
