# Supplementary material for: Understanding factors associated with rural‐urban disparities of stunting among under‐five children in Rwanda: A decomposition analysis approach
Source: Matern Child Nutr. 2023 Mar 30;19(3):e13511. doi: 10.1111/mcn.13511 (PMC10262907; doi:10.1111/mcn.13511)

Supplementary file 4 Figure 1: A decomposition analysis of factors contributing to severe stunting reduction in rural areas

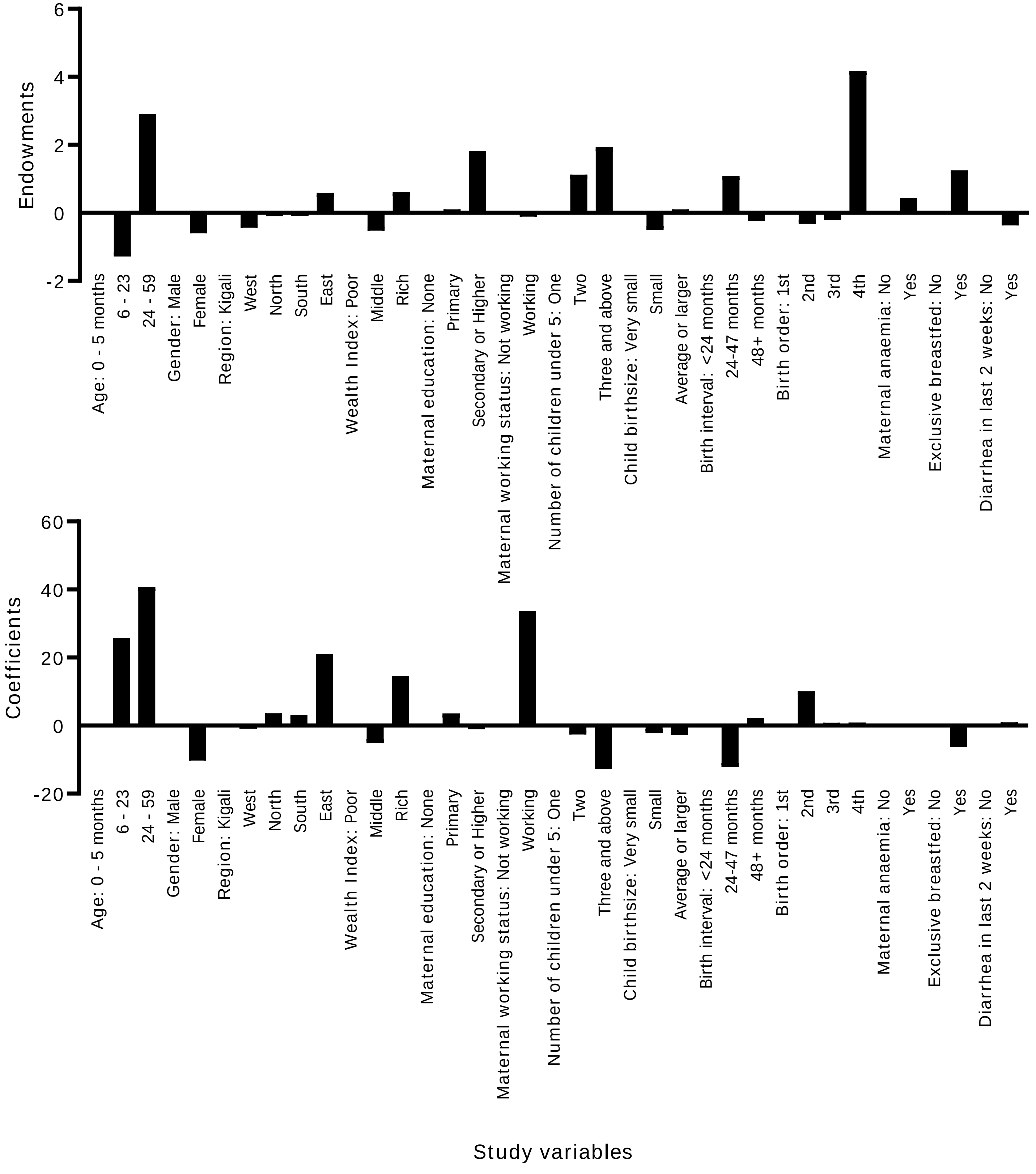

Supplementary file 4 Figure 2: A decomposition analysis of factors contributing to severe stunting reduction in urban areas

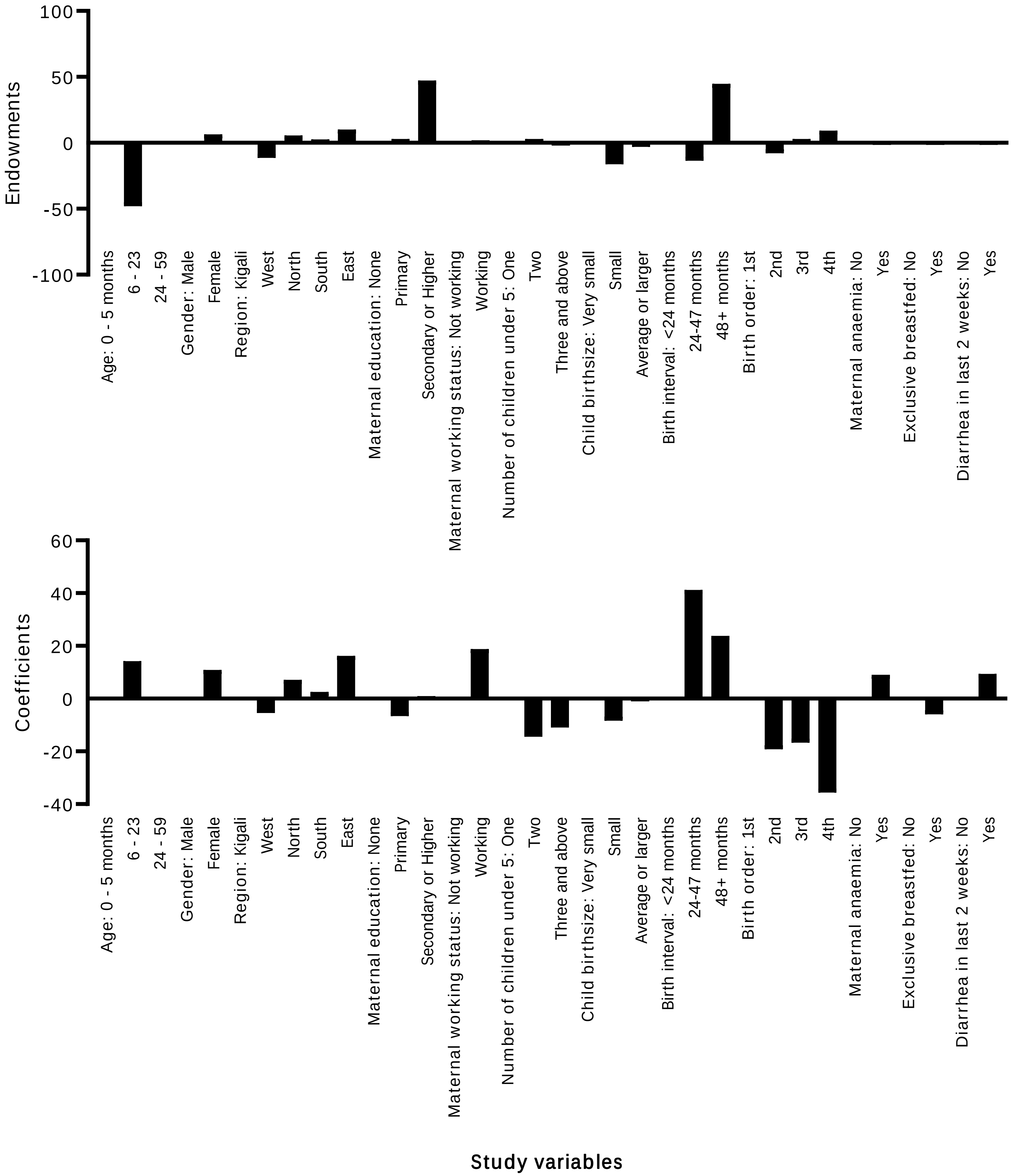

Supplement: Supplementary file 4 — Supporting information. [file MCN-19-e13511-s003.pdf]
